# Phosphatase of regenerating liver-3 is expressed in acute lymphoblastic leukemia and mediates leukemic cell adhesion, migration and drug resistance

**DOI:** 10.18632/oncotarget.23186

**Published:** 2017-12-13

**Authors:** Magnus A. Hjort, Pegah Abdollahi, Esten N. Vandsemb, Mona H. Fenstad, Bendik Lund, Tobias S. Slørdahl, Magne Børset, Torstein B. Rø

**Affiliations:** ^1^ Department of Clinical and Molecular Medicine, Norwegian University of Science and Technology, Trondheim, Norway; ^2^ Children’s Clinic, St. Olavs Hospital, Trondheim University Hospital, Trondheim, Norway; ^3^ Department of Immunology and Transfusion Medicine, St. Olavs Hospital, Trondheim University Hospital, Trondheim, Norway; ^4^ Department of Hematology, St. Olavs Hospital, Trondheim University Hospital, Trondheim, Norway

**Keywords:** PRL-3, acute lymphoblastic leukemia, migration, adhesion, drug resistance

## Abstract

Phosphatase of regenerating liver-3 *(PRL-3/PTP4A3)* is upregulated in multiple cancers, including BCR-ABL1- and ETV6-RUNX-positive acute lymphoblastic leukemia (ALL). With this study, we aim to characterize the biological role of PRL-3 in B cell ALL (B-ALL). Here, we demonstrate that PRL-3 expression at mRNA and protein level was higher in B-ALL cells than in normal cells, as measured by qRT-PCR or flow cytometry. Further, we demonstrate that inhibition of PRL-3 using shRNA or a small molecular inhibitor reduced cell migration towards an SDF-1α gradient in the preB-ALL cell lines Reh and MHH-CALL-4. Knockdown of PRL-3 also reduced cell adhesion towards fibronectin in Reh cells. Mechanistically, PRL-3 mediated SDF-1α stimulated calcium release, and activated focal adhesion kinase (FAK) and Src, important effectors of migration and adhesion. Finally, PRL-3 expression made Reh cells more resistance to cytarabine treatment. In conclusion, the expression level of PRL-3 was higher in B-ALL cells than in normal cells. PRL-3 promoted adhesion, migration and resistance to cytarabine. PRL-3 may represent a novel target in the treatment of B-ALL.

## INTRODUCTION

Acute lymphoblastic leukemia (ALL) is the most common childhood cancer, accounting for approximately one third of pediatric cancers. Major prognostic factors include clinical features at time of diagnosis, and biological and genetic features of leukemic cells [1]. The overall prognosis in children has improved substantially over the last decades, and today the 5-year survival in Western countries has reached 90 %. However, ALL is still the most frequent cause of death from cancer in children, mainly due to a poor prognosis at relapse [1]. Thus, identification of novel targets for treatment as well as reduction of treatment-related toxicity are still important for increased survival [2].

Adhesion to bone marrow (BM) stroma and BM niches is vital for both normal and leukemic B-progenitor cells [3, 4]. Adhesion of B cells to BM stroma is achieved primarily through binding via the beta-1 integrin α_4_β_1_ (very late antigen (VLA)-4) and α_5_β_1_ (VLA-5) [5, 6]. Adhesion enables the cells to receive growth and survival signals, either through integrin-mediated signaling or through stimulation by interleukin (IL)-7 and other cytokines in the bone marrow microenvironment [7]. Integrins are also necessary for the migration of progenitor cells through stromal layers, a process regulated by stromal cell-derived factor-1 alpha (SDF-1ɑ) [8]. During cancer therapy, cell adhesion in the BM promotes resistance to chemotherapy [5, 6]. This cell adhesion-mediated drug resistance (CAM-DR) has been considered a cause of minimal residual disease (MRD) and relapse of ALL [6].

PRL-3 is a dual-specificity phosphatase, meaning that it can dephosphorylate both tyrosine and serine/threonine residues, and is encoded by the gene *PTP4A3*. PRL-3 was first described in 1998, and associated with metastatic potential in colorectal cancer [9]. Today, PRL-3 is known to be important for cancer cell motility, adhesion, invasion and metastasis in several solid tumors [10]. In hematological cancers, less is known about the role of PRL-3. We have previously shown that *PRL-3* is overexpressed in cancer cells from patient with multiple myeloma (MM), compared to normal plasma cells [11]. An oncogenic role of PRL-3 is described in acute myeloid leukemia (AML) [12–16] and chronic myeloid leukemia [17].

PRL-3 mRNA is highly expressed in BCR-ABL ALL [18], and was found overexpressed in ETV6-RUNX1 ALL, but did not impact cell viability [19]. Based on the oncogenic role and expression of PRL-3 in other hematological cancer, we aimed to explore PRL-3’s role in the pathogenesis of B-ALL.

## RESULTS

### PRL-3 was expressed in higher levels in B-ALL patient cells than in healthy control cells

**Figure 1:**
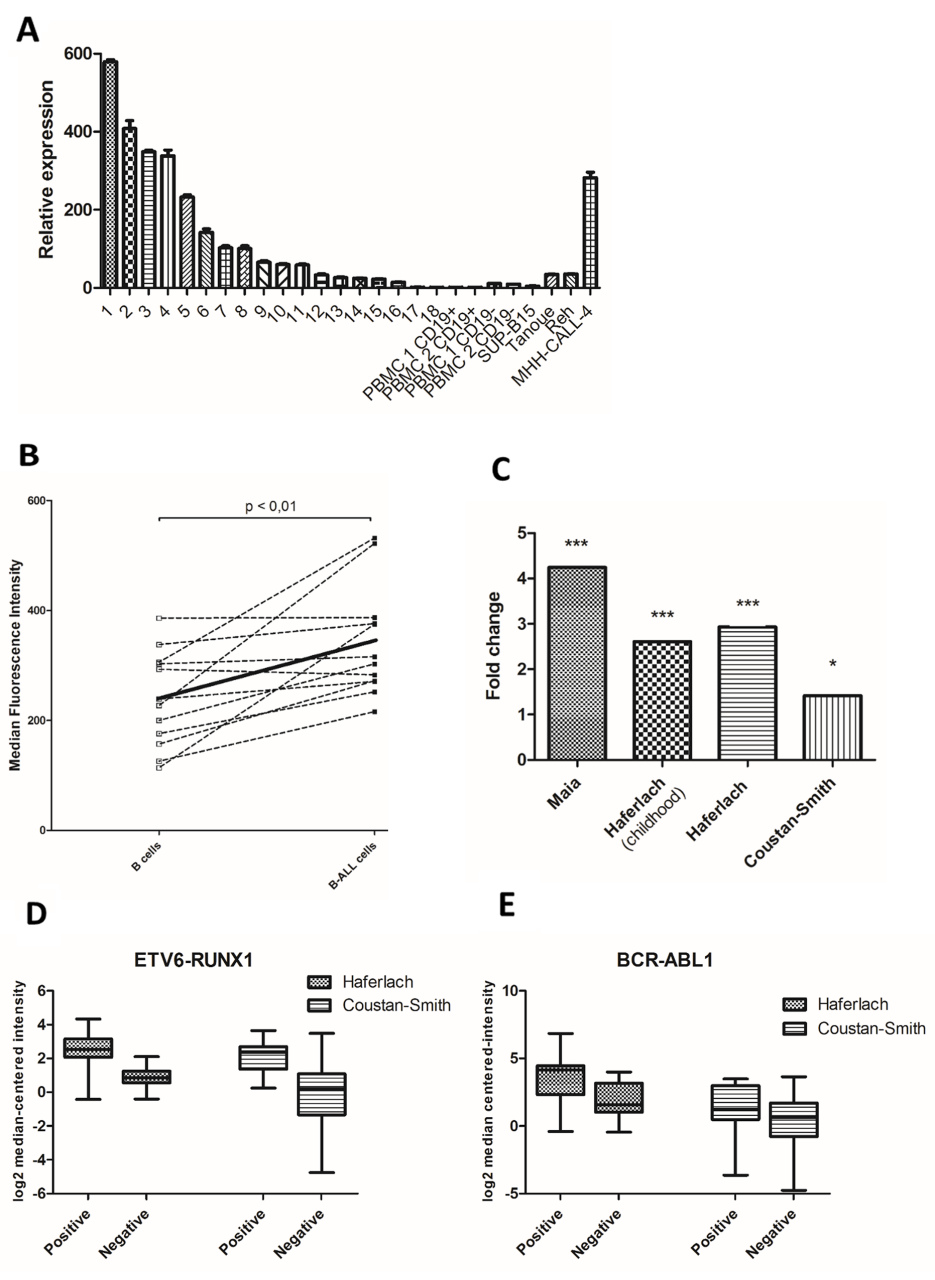
PRL-3 was expressed in higher levels in B-ALL patient cells than in healthy control cells **(A)** Figure represents relative *PRL-3* expression of 18 adult B-ALL patients, CD19+ and CD19-separated cells from 2 healthy controls and four cell lines. Samples were normalized to their GAPDH level (2^-ΔΔCt^ method), and *PRL-3* expression in patient 18 was set to 1 (The ΔCt between PRL-3 and GAPDH for patient 18 was 11,0 (± 0,12)). *Error bars* represents + 1 SD of triplicates. Clinical data are shown in Supplementary Table 1. **(B)** In a consecutive examination of children with B-ALL, at the time of diagnosis, B-ALL cells expressed higher PRL-3 than normal B cells measured by flow cytometry. Values represent median fluorescence intensity (MFI) for each patient. The solid line indicates mean value for PRL-3 in normal and leukemic B cells. The mean value was significantly higher in B-ALL cells compared to normal cells (p<0.01). Clinical data are shown in Supplementary Table 2. **(C)** In three available datasets in the Oncomine database, *PRL-3* was significantly overexpressed in B-ALL patients compared to normal control. Each bar represents fold change of *PRL-3* in B-ALL samples compared to normal control. Further information of the datasets is shown in Table 1. ^*^ indicates p < 0,05, ^***^ indicates p < 0,001. Two of the datasets, Haferlach and Coustan-Smith, provided ETV6-RUNX1- and BCR-ABL1-status. Dividing the datasets in ETV6-RUNX1- **(D)** and BCL-ABR1- **(E)** positive or negative, there was higher expression of *PRL-3* in the positive group for both translocations. Still, translocation-negative samples expressed *PRL-3*. Line in the middle of the box represents median and boxes represents 25-75 percentiles of log2 median-centered intensity of *PRL-3* expression. Whiskers represents minimum and maximum value. Normal samples and other leukemic samples are not shown. Data adapted from Oncomine.

First, we investigated *PRL-3* mRNA expression in 18 adult B-ALL patient samples, peripheral mononuclear cells (PBMC) from two healthy controls and four B-ALL cell lines. Patient characteristics are shown in Supplementary Table 1. The expression level varied 600-fold between samples (Figure 1A). There were no significant differences in overall survival, relapse, white blood cell count or percentage of blasts in peripheral blood between patients with expression of *PRL-3* higher and lower than the median expression (data not shown). To assess PRL-3 protein expression, we measured PRL-3 in BM from 12 children with B-ALL at the time of diagnosis using flow cytometry. Clinical data are shown in Supplementary Table 2. PRL-3 was significantly higher expressed in the leukemic B cells than in the individual patients’ normal B cells (p < 0,01) (Figure 1B). A search for “PTP4A3”, “B cell ALL” and “Cancer Vs. Normal Analysis”, in the open online gene expression profiling source www.oncomine.org, identified three datasets [20–22]. *PRL-3* expression was significantly higher in B-ALL samples than in normal samples in all three datasets (Figure 1C and Table 1). Two of the datasets [21, 22], provided ETV6-RUNX1 and BCR-ABL1 status. Dividing these data into positive or negative ETV6-RUNX1 (Figure 1D) or BCR-ABL1 (Figure 1E), resulted in higher *PRL-3* expression in the translocation positive group for both conditions. Collectively, this indicates that PRL-3 is overexpressed in B-ALL cells compared to normal B cells.

**Table 1:** Datasets obtained from www.oncomine.org

Datasets	B-ALL samples	Control samples	Fold change	p-value	Reference
**Maia Leukemia (childhood)**	20	7 (Immature B-lymphocyte and Pre B-Lymphocyte)	4,24	2,45^*^10^-11	[20]
**Haferlach Leukemia (childhood)**	359	74 (PBMC)	2,61	2,18^*^10^-66	[21]
**Haferlach Leukemia**	147	74 (PBMC)	2,93	2,72^*^10^-38	[21]
**Coustan-Smith Leukmia (childhood)**	238	4 (CD10+ and CD19+ Hematogone)	1,42	0,031	[22]

PBMC - Peripheral blood mononuclear cells.

### PRL-3 was induced by cytokines in B-ALL cell lines

**Figure 2:**
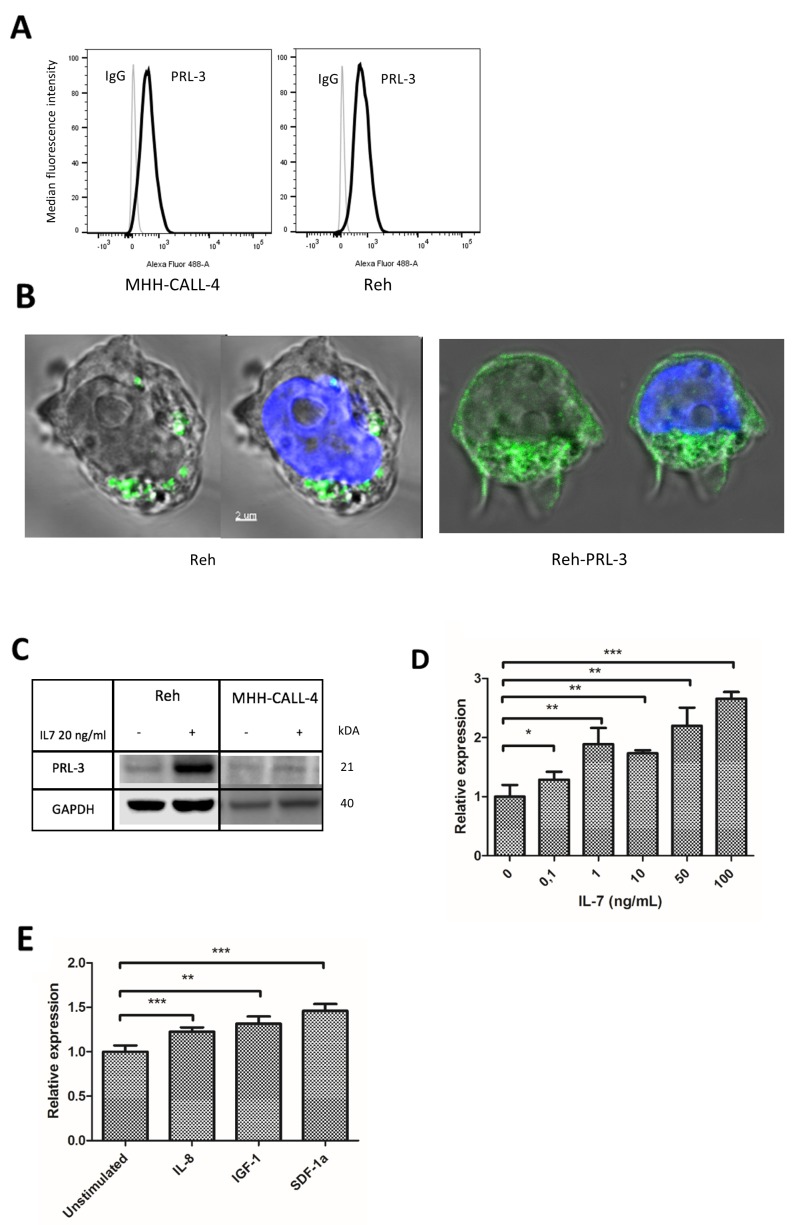
PRL-3 was induced by cytokines in B-ALL cell lines **(A)** PRL-3 expression was detected by flow cytometry in Reh and MHH-CALL-4. Median fluorescence intensity value is normalized. **(B)** By confocal microscopy, PRL-3 was detected as punctuate structures in the cytoplasm of Reh cells. In Reh overexpressing PRL-3 cells, PRL-3 was also detected in the plasma membrane. Green is anti-PRL-3 and blue is DNA/nucleus. **(C)** PRL-3 protein was determined by Western analysis in Reh and MHH-CALL-4 cells stimulated by IL-7 20 ng/mL for 48 hours. Stimulation with IL-7 increased PRL-3 protein in Reh cells. **(D)** IL-7 induced *PRL-3* expression in a dose-dependent manner.*PRL-3* mRNA was quantified by qRT-PCR after Reh cells were stimulated by IL-7 for 48 hours. The relative expression level of *PRL-3* in unstimulated cells was arbitrarily set to 1, and samples were normalized to their GAPDH level (2^-ΔΔCt^ method). *Error bars* represents + 1 SD of triplicates. **(E)** IL-8, IGF-1 and SDF-1α (all 100 ng/mL) were able to induce mRNA expression of *PRL-3*. *Error bars* represents + 1 SD of triplicates. ^*^ indicates p < 0,05. ^**^ indicates p < 0,01. ^***^ indicates p < 0,001.

To explore biological and molecular mechanism of PRL-3 in B-ALL, we chose to use the PreB-ALL cell lines Reh and MHH-CALL-4, which express moderate to high PRL-3 mRNA levels for further experiments (Figure 1A). PRL-3 protein expression is not always correlated with gene expression. We found PRL-3 protein present in Reh and MHH-CALL-4 cell lines as examined by intracellular flow cytometry (Figure 2A), by confocal microscopy (Figure 2B) and WB (Figure 2C). Because Reh cells expressed more PRL-3 protein than MHH-CALL-4, we focused subsequent experiments on Reh cells. Next, we examined whether PRL-3 could be induced by growth-inducing cytokines produced in the BM microenvironment. After 48 hours of stimulation with IL-7, there was an increase in protein expression in Reh, and to some extent in MHH-CALL-4 cells (Figure 2C). IL-7 induced a significant dose-dependent increase in *PRL-3* mRNA in Reh (Figure 2D). Stimulation with IL-8, IGF-1 and SDF-1ɑ increased *PRL-3* expression significantly in Reh by 23 %, 32 % and 46 % respectively (Figure 2E).

### PRL-3 mediated migration and adhesion of B-ALL cell lines

**Figure 3:**
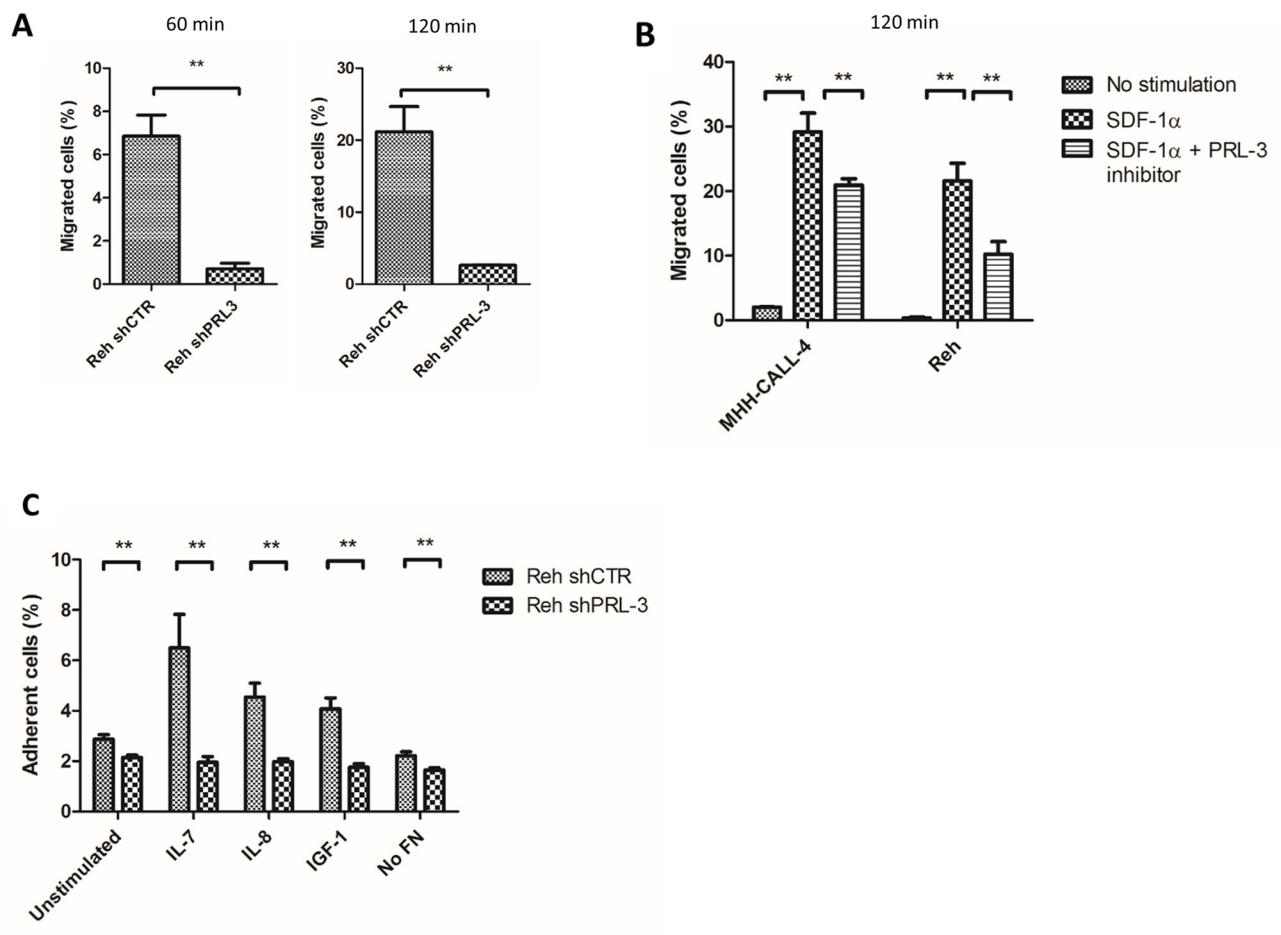
PRL-3 mediated migration and adhesion of B-ALL cell lines **(A)** Knockdown of PRL-3 by shRNA in Reh cells reduced the migration towards a SDF-1ɑ gradient by 90 and 88 % after 60 (left) and 120 (right) minutes, respectively. Cells were seeded in the upper well of a transwell migration assay. SDF-1ɑ (75 ng/mL) was added in the lower compartment. Cells that migrated to the lower compartment were counted. Bars represent the percentage of migrated cells from one representative of three independent experiments. *Error bars* represent + 1 SD of three repeated counts in two parallels. **(B)** PRL-3 inhibitor I reduced SDF-1ɑ mediated migration in Reh and MHH-CALL-4 with 53 % and 28 % respectively. *Error bars* represent + 1 SD of three repeated counts in two parallels from one representative of three independent experiments. **(C)** Cytokine induced adhesion to FN is mediated by PRL-3. Reh shCTRL cells adhered significantly more to FN than Reh shPRL-3. Bars represents the percentage of adherent cells (+ 1 SD) of six samples from one representative of three independent experiments. ^**^ indicates p < 0,01. ^***^ indicates p < 0,001.

Previous studies have demonstrated a role for PRL-3 in the adhesion and migration of cancer cells. To examine PRL-3’s effect on migration of leukemic B cells, we first knocked down PRL-3 in Reh cells using PRL-3 shRNA (Supplementary Figure 1A). In a Transwell migration assay, addition of SDF-1ɑ (75 ng/mL) to the lower compartment gave a good migratory response as expected (Figure 3A–3B). Knockdown of PRL-3 reduced SDF-1ɑ-induced migration by 90 and 88 % compared to control cells after 60 and 120 minutes, respectively (Figure 3A). To confirm these results, we wanted to see whether the small molecular compound, PRL-3 inhibitor I, could inhibit migration. The inhibitor (40 μM) reduced the number of migrated cells in both Reh and MHH-CALL-4 cells, with 53 % and 28 %, respectively (Figure 3B). The reduction in migration was not caused by anti-apoptotic effects of the PRL-3 inhibitor in Reh or MHH-CALL-4 as determined by annexin-V and PI (Supplementary Figure 2). These results indicate that PRL-3 mediated the SDF-1ɑ-induced migration of B-ALL cells.

Adhesion of leukemic cells to stroma is important for cell renewal and can promote drug resistance. To examine cell adhesion, we incubated Reh shCTRL and Reh shPRL-3 cells in wells coated with fibronectin (FN) (Figure 3C). Stimulation with IL-7, IL-8 and IGF-1 increased adhesion 2.5-3-fold in Reh shCTRL cells, whereas there was no increase in adhesion in Reh shPRL-3 cells (Figure 3C). These results indicate that PRL-3 is necessary for cytokine stimulated adhesion of leukemic cells to FN.

### Mechanism for PRL-3-induced migration and adhesion

To elucidate possible mechanisms for the effects of PRL-3 on migration and adhesion, we looked into downstream targets of SDF-1ɑ signaling involved in migration and adhesion including focal adhesion complex and Ca^2+^ mobilization.

#### Ca^2+^ mobilization

**Figure 4:**
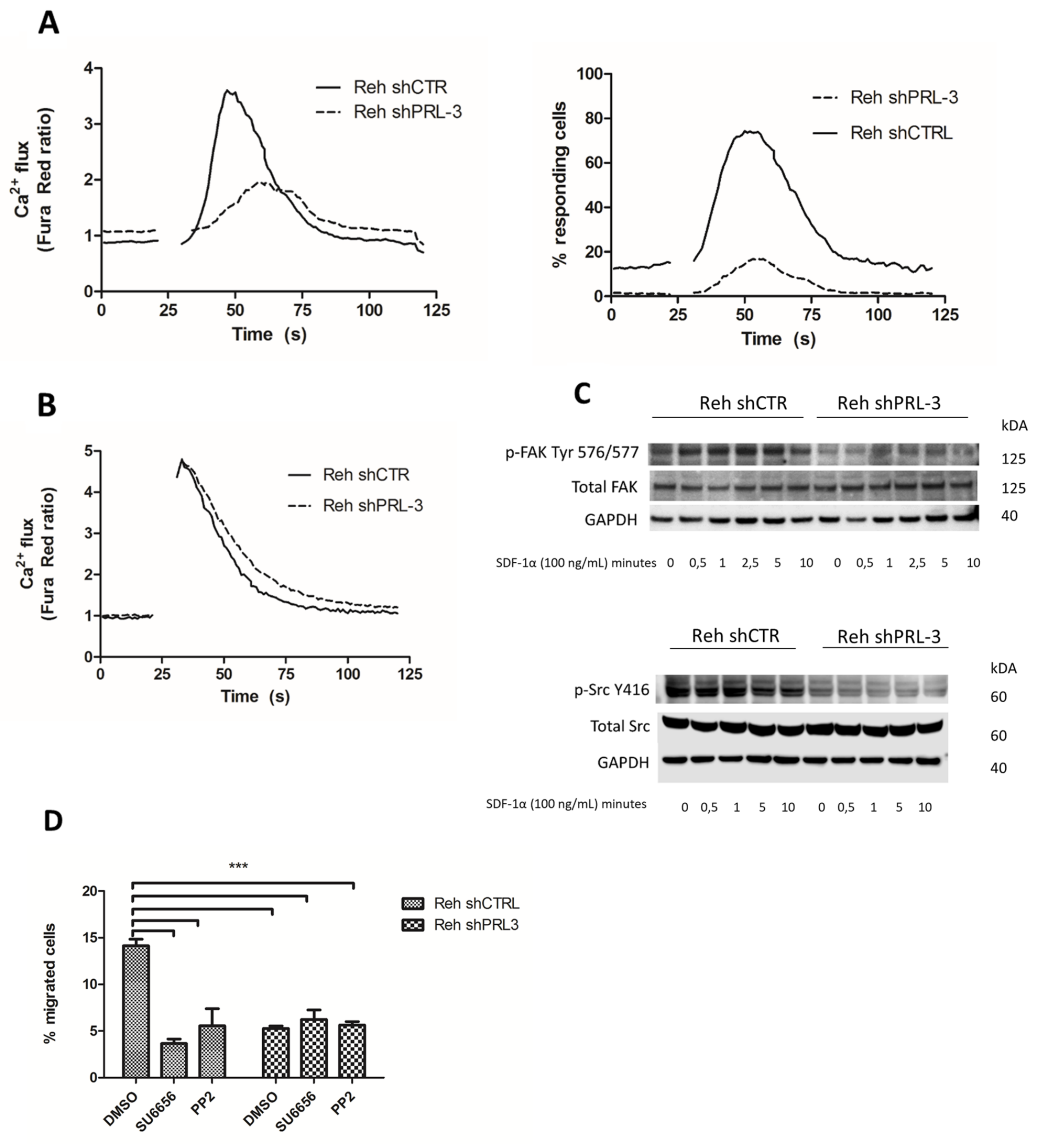
PRL-3 mediated calcium release, and increased pSrc-Y 416 and pFAK-Y576/577 as a possible mechanism for mediating migration and adhesion **(A)** SDF-1α stimulated release of calcium, and number of responding cells, was higher in the presence of PRL-3. **(B)** CSK stimulated calcium release was independent of PRL-3 expression. Gap indicates time used for removal of tube for adding stimulus. Background signal was recorded for 20 seconds, the sample was then removed and stimulated with SDF-1ɑ (100 ng/mL) or CSK (1:500), and returned for continuing recording for total of 120 seconds. **(C)** SDF-1α increased the phosphorylation of FAK Tyr 576/577 in Reh shCTRL. Phosphorylation of Src was also increased in Reh shCTRL compared to PRL-3 knockdown cells, but was not induced by SDF-1α stimulation. Total Src and FAK was unchanged by PRL-3 knockdown. The membranes were reprobed for total Src or FAK, and GAPDH. **(D)** Src inhibition removed the PRL-3 effect of SDF-1ɑ (75 ng/mL) induced migration. Bars represent the percentage of migrated cells from one representative of three independent experiments. *Error bars* represent + 1 SD of three repeated counts in two parallels. ^***^ indicates p < 0,001.

SDF-1ɑ is known to induce a rapid Ca^2+^ flux in B-ALL cells, which can be measured using Fura Red. In the Reh shCTRL cells, there was a rapid increase in signal with a peak almost 4-fold above baseline after stimulation with SDF-1α, indicating an increase in Ca^2+^ flux. Knockdown of PRL-3 resulted in both slower response and lower amplitude of the Ca^2+^ signal, with the peak only 1,6-fold above baseline (Figure 4A). The percentage of responding cells was also lower in the PRL-3 knockdown cells (Figure 4A). Stimulation with the positive control, CSK, induced the same Ca^2+^ flux in both cell lines (Figure 4B). Collectively, this indicates that PRL-3 amplifies the release of Ca^2+^ induced by the receptor for SDF-1ɑ (CXCR4).

#### Focal adhesion complex

FAK and Src are key components in mediating migration and adhesion, and can be activated by SDF-1ɑ signaling. By Western blotting, we found that Reh shCTRL cells expressed more activated pFAK-Tyr576/577 than Reh knockdown cells (Figure 4C). Stimulation of Reh shCTRL cells with SDF-1ɑ increased phosphorylation of FAK within 30 seconds, with a peak after 2.5 minutes. No such increase in FAK phosphorylation was seen in Reh PRL-3 knockdown cells (Figure 4C). The basal level of pSrcY-416 was higher in Reh control cells compared to Reh shPRL-3 cells, and did not increase with stimulation with SDF-1ɑ (Figure 4C). Finally, we wanted to test whether the PRL-3-mediated effect of migration was caused by Src activation. Treating Reh shCTRL and Reh shPRL3 with two different Src inhibitors (SU6656 or PP2), before stimulation with SDF-1ɑ in a transwell system, removed the PRL-3 effect on migration (Figure 4D).

The CXCR4 mRNA expression and surface expression, and surface expression of beta-1 integrin/CD29 were unchanged following PRL-3 knockdown (data not shown). Taken together, this suggests that PRL-3 facilitates SDF-1ɑ-induced Ca^2+^ mobilization and activation of FAK, and in the absence of SDF-1ɑ, increases activation of FAK and Src.

### Drug resistance

**Figure 5:**
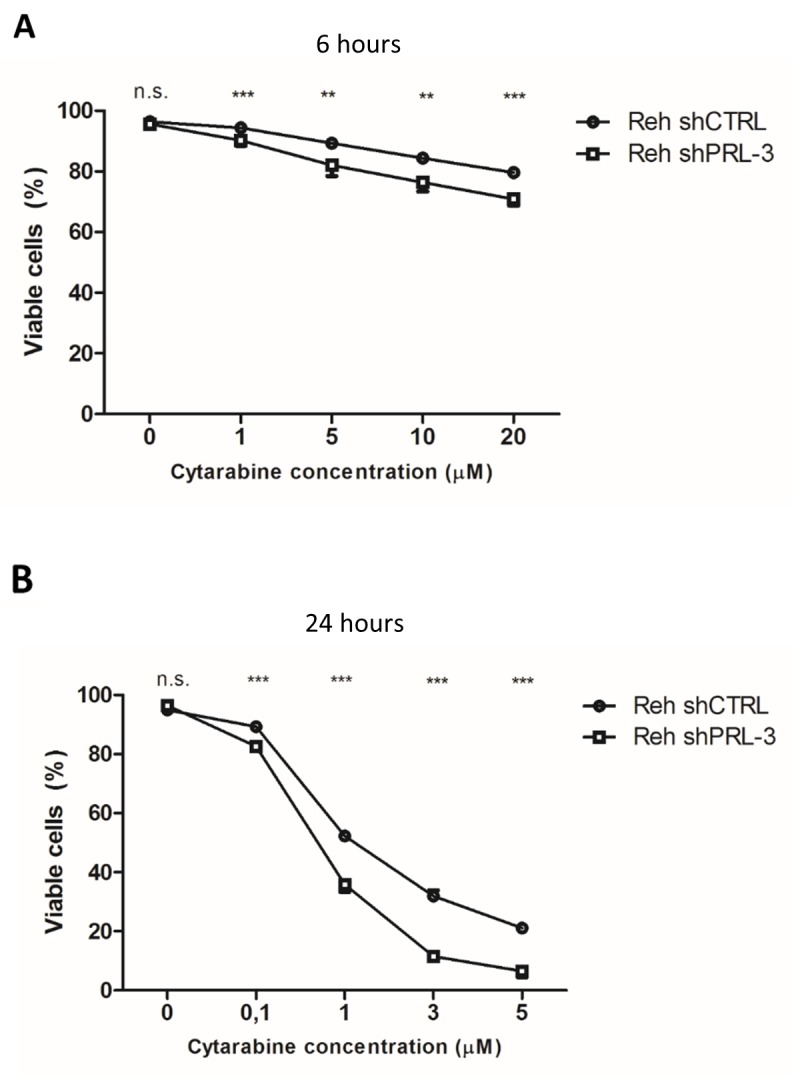
PRL-3 protects Reh cells from apoptosis induced by cytarabine after **(A)** 6 hours and **(B)** 24 hours. *Error bars* represent + 1 SD of two independent experiments with duplicates. Viability was determined by annexin V-FITC/propidium iodide flow cytometry. ^**^ indicates p < 0,01. ^***^ indicates p < 0,001.

Finally, we screened for resistance to common anti-leukemic drugs caused by PRL-3. With cytarabine treatment, for 6 and 24 hours, we found that Reh shCTRL cells were significantly more viable compared to Reh shPRL-3 cells (Figure 5A–5B). We did not find any difference in viability after treatment with prednisone, daunorubicin or vincristine (data not shown).

## DISCUSSION

In this study, we describe a possible role for PRL-3 in the pathogenesis of B-ALL. We show that *PRL-3* was expressed in B-ALL patient samples and cell lines. Further, we found that PRL-3 was overexpressed, at both mRNA and protein level, in B-ALL cells compared to healthy control cells. Importantly, via generation of PRL-3 knockdown cells, we have shown that PRL-3 supports leukemic cell adhesion, migration and drug resistance. Collectively, this points to PRL-3 as a possible novel target in B-ALL.

We detected *PRL-3* in most of our B-ALL samples, and in the Oncomine datasets *PRL-3* was significantly higher expressed in B-ALL cells compared to normal control cells. ETV6-RUNX1- and BCR-ABL1-positive cases had a higher *PRL-3* expression compared to negative samples, as also shown by others [18, 19]. Still, even translocation-negative samples expressed *PRL-3*. As shown by flow cytometry, leukemic cells had a significantly higher expression of PRL-3 compared to normal B cells from the same patient at the time of diagnosis. Overexpression of PRL-3 in B-ALL cells is in accordance with previous studies of PRL-3 expression in cancer tissues. In normal tissue, PRL-3 expression is generally low after the fetal period. PRL-3 mRNA can be found in heart and skeletal muscle, and at low levels in other tissues, but PRL-3 protein expression is rarely demonstrated [10, 23]. In hematological cells, *PRL-3* is expressed at low levels in megakaryocyte-erythroid progenitor cells, but not detected otherwise in bone marrow or in lymph nodes [23, 24]. PRL-3 has previously been reported to be expressed in hematological cancers like AML, MM [11–16], and based on our findings PRL-3 is also overexpressed in B-ALL.

We show that knockdown of PRL-3 with shRNA or inhibition of PRL-3 with PRL-3 inhibitor I reduced SDF-1α-mediated migration, indicating a mechanistic role of PRL-3 in leukemic cell migration. Knockdown of PRL-3 also reduced cytokine-stimulated adhesion of leukemic cells to FN. Motility and adhesion are important in the pathogenesis of cancer. SDF-1α stimulates leukemic cells to migrate through stromal layers, enabling them to gain access to niches normally restricted to progenitor cells, and thereby favoring growth and survival [25]. Adhesion of leukemic cells within niches in the BM has been hypothesized to be a mechanism for relapse and drug resistance through CAM-DR [5, 6, 26]. Since the very first paper on PRL-3, showing high expression of PRL-3 in liver metastases, but not in the primary colon tumor cells, there have been several reports describing a role for PRL-3 in migration and motility of cells [27]. In our experiments, knocking down PRL-3 was enough to almost completely abrogate migration, making PRL-3 an attractive target for novel therapies.

SDF-1α signaling can activate FAK and Src, key components of focal adhesions complexes [28–30]. FAK integrates external signals to promote cell motility via different pathways involving Src, p130Cas, MAPK, guanine exchange factors (GEFs) and Rho-family GTPases [31]. Many of these are shown to be affected by PRL-3 [27]. Activation of FAK causes auto-phosphorylation at Tyr 397, which act as a binding site for Src, leading to phosphorylation of Tyr 576/577. Zimmerman *et al* found that absence of PRL-3 in endothelial cells, created from PRL-3-null mice, resulted in less phosphorylation of Src and FAK [32]. We have recently shown that PRL-3 regulates Src activation in MM cells by increasing the phosphorylation of Src-Y416 [33], thus activating the oncogenic kinase. Similar to our present findings, Gari *et al* found that inhibition of PRL-3 by shRNA or an inhibitor (Ampi-109), inactivated Src by reducing the phosphorylation of Y416 in triple-negative breast cancer [34]. Blocking Src reduced the PRL-3-mediated effect on migration in our experiments. This, together with reduced Src activation in PRL-3 knockdown cells, indicates that Src activation is downstream of PRL-3. Taken together, our present study demonstrates that PRL-3 expression is necessary for SDF-1ɑ induced migration B-ALL cells, presumably mediated through activation of Src and FAK.

The SDF-1α receptor (CXR4) is a G protein-coupled receptor, and calcium flux is one of the first responses after ligand binding [35]. Calcium flux is used as a measure for activation of the CXCR4-receptor. SDF-1α signaling further increase phosphorylation of focal adhesion components, including FAK [28]. Interestingly, we found that knockdown of PRL-3 impaired Ca^2+^ flux induced by CXCR4 activation, but not that induced by CSK. This indicates that PRL-3 is permissive for Ca^2+^ release by SDF-1α. The opposite was described in normal HEK293 cells, where overexpression of PRL-3 inhibited angiotensin-II stimulated Ca^2+^ flux [36]. Whether this is a cell-dependent, cancer- or cytokine-specific function of PRL-3 is unknown.

Drug resistance is a major cause of relapse of ALL. We show here that PRL-3 expression made Reh cells more resistant to cytarabine. Cytarabine is an antimetabolite prodrug. It is transported into cells through hENT1, and converted into active form, cytosine arabinoside triphosphate, by phosphorylation by deoxycytidine kinase (dCK) [37]. Whether PRL-3, as a phosphatase, is directly involved in inhibition the transportation, the conversion to the active form, or another mechanism, needs to be further explored. For the other drugs, we did not observe any difference in viability, which is in accordance with others [19].

## CONCLUSION

We found that PRL-3 mRNA and protein was expressed in B-ALL patient samples and cell lines, and overexpressed in patient samples compared to normal controls. Knockdown of PRL-3 impeded both migration and adhesion of B-ALL cells *in vitro*, presumably through regulation of Ca^2+^ flux and phosphorylation of FAK and Src. Finally, PRL-3 expressing cells were more resistant to cytarabine treatment. This indicates that PRL-3 may represent a novel target for treatment of B-ALL.

## MATERIALS AND METHODS

### Cell cultures and patient samples

We used human B-ALL cell lines Reh (ATCC, Rockville, MD), MHH-CALL-4, Tanoue and SUP-B15 (DSMZ, Brauschweig, Germany). Cells were grown in RPMI-1640 supplemented with 2 mmol/L l-glutamine, 40 μg/mL gentamicin, and 10 % (Reh and Tanoue) or 20 % (MHH-CALL-4 and SUP-B15) heat-inactivated FCS (hereafter called medium). New cell cultures were seeded every 4 months from frozen vials aliquoted shortly after receiving the cells from the original supplier. Cells were cultured at 37°C in a humidified atmosphere with 5 % CO_2_, and growth medium were replenished twice weekly. Frozen isolated peripheral blood mononuclear cells (PBMC) from B-ALL patients were provided by The Regional Biobank of Central Norway and fresh BM samples were obtained from children admitted to St. Olavs hospital, both after obtaining informed consent. PBMC from healthy control were obtained from Department of Immunology and Transfusion Medicine, St. Olavs hospital. PBMC were separated in CD19+ and CD19- cells using CD19 MicroBeads (Miltenyi Biotec GmbH, Bergisch Gladbach, Germany) according to manufacturer’s instructions.

### Antibodies, cytokines and other reagents

Recombinant human IL-7, IL-8 and SDF-1α were purchased from Peprotech (Rocky Hill, NJ), and insulin-like growth factor 1 (IGF-1) was from R&D Systems (Abingdon, UK). Antibody against PRL-3 (318) (#sc-130355), Alexa 488-conjugated anti-PRL-3 (318) and control mouse IgG_1_-Alexa Fluor® 488 (#sc-3890) were bought from Santa Cruz Biotechnology (Santa Cruz, TX). Antibody against GAPDH was from Abcam (Cambridge, United Kingdom), and antibodies against phospho-focal adhesion kinase (pFAK) (Tyr576/577) (#3281), total FAK (#3285), pSrc (Y416) (#2101) and total Src (#2109) were from Cell Signaling Technology (Beverly, MA). PE-labelled anti-human CD184 (C-X-C chemokine receptor type 4 (CXCR4)), PE-labelled anti-active human CD29 (HUTS-21) and irrelevant mouse IgG_1_ and IgG_2_ antibodies were from Pharmingen (Bedford, MA). Human plasma fibronectin (FN) was from BD Biosciences (Bedford, MA). PRL-3 inhibitor I (5-[[5-Bromo-2-[(2-bromophenyl)methoxy]phenyl]methylene]-2-thioxo-4-thiazolidinone), SU6656 (2,3-Dihydro-N,N-dimethyl-2- oxo-3-[(4,5,6,7-tetrahydro-1H-indol-2-yl)methylene]-1H-indole- 5-sulfonamide), prednisolone, vincristine and daunorubicin were bought from Sigma-Aldrich (St. Louis, MO). PP2 Src inhibitor (4-Amino-3-(4-chlorophenyl)-1-(t-butyl)-1H-pyrazolo[3,4-d] pyrimidine, 4-Amino-5-(4-chlorophenyl)-7-(t-butyl)pyrazolo[3, 4-d] pyrimidine) was from Santa Cruz Biotechnology. The plasmids pLKO (non-silencing control) and shRNA-pLKO against PRL-3 were a kind gift from Dr. Jim Lambert (University of Colorado, Denver, CO) [34].

### Lentiviral transduction for PRL-3 knockdown and overexpression

For stable PRL-3 knockdown 293T packaging cells were transfected with individual pLKO-shRNA against PRL-3 or pLKO (control plasmid) in combination with psPAX2 (packaging plasmids) and pMD2.G (envelope plasmid) for virus production. Reh cells were transduced with viruses produced by packaging cells in order to establish Reh PRL-3 knockdown (Reh shPRL-3) and control cell line (Reh shCTRL). Knockdown was confirmed by qRT-PCR and intracellular flow cytometry (Supplementary Figure 1A-1B).

To establish cells with doxycycline (1 μg/mL)-inducible PRL-3, 293T packaging cells were transfected with PCW57.1 (control plasmid) (AddGene, Cambridge, MA) and PRL3-PCW57.1 (plasmid containing the human PTP4A3 ORF cDNA) in combination with psPAX2 (packaging plasmids) and pMD2.G (envelope plasmid) for virus production. The PRL-3-PCW57.1 was made by performing an LR recombination reaction between the ORF PTP4A3 cDNA clone: ORFEXPRESS Gateway PLUS shuttle clone (GC-Z7908; GeneCopoeia, Rockville, USA) and the PCW57.1 plasmid. Reh cells were transduced with viruses produced by packaging cells in order to establish inducible overexpressing PRL-3 (Reh-PRL-3) and empty control cell line (Reh-Mock). Transduced cells were selected in medium with puromycin (0,5 μg/mL).

### Immunoblotting

Cells were treated as indicated, then pelleted, lysed and immunoblotted as previously described [38]. Images were acquired using LI-COR Odyssey Fc and analyzed with Image Studio Software (LI-COR, Lincoln, Nebraska). All experiments were conducted at least three times.

### RNA isolation, cDNA synthesis and quantitative real time PCR (qRT-PCR)

Isolation of RNA and cDNA synthesis were performed as previously described [39]. For relative quantification, we used the comparative 2^-DDCT^-method, with GAPDH as endogenous reference. All samples were run in triplicate. The following primers were used: PTP4A3 (Hs02341135_m1), CXCR4 (Hs00607978_s1) and GAPDH (Hs99999905_m1) (TaqMan). Analysis was performed using Step One Software 2.3 (Applied Biosystems).

### Apoptosis assay

Viability and apoptosis were evaluated using Annexin V-FITC binding and propidium iodide (PI) uptake (APOPTEST-FITC kit; Nexins Research, Kattendijke, The Netherlands). Briefly, cells were washed once with ice-cold PBS, resuspended and incubated on ice for 1 hour in the dark in 300 μL binding buffer with 0,25 μL annexin V-FITC. PI was added 5 minutes before the samples were analyzed by flow cytometry using BD LSRII Flow Cytometer (BD Biosciences, Franklin Lakes, NJ). All conditions were run in duplicate and repeated three times.

### Migration assay

Cells (4^*^10^5^) were seeded into the upper chamber of a Transwell culture plate (polycarbonate membrane with pore size 3 μm, Corning, NY). In experiments using inhibitors, cells were pre-treated with or without PRL-3 inhibitor I (40 μM) for 30 minutes, SU6656 (5 μM) or PP2 (10 μM) for 4 hours before seeded into the upper chamber. The lower chamber was loaded with 600 μL medium with SDF-1α (75 ng/mL) as a chemoattractant, and PRL-3 inhibitor I (40 μM), SU6656 (5 μM) or PP2 (10 μM) as indicated. The cells that migrated through the membrane to the lower chamber were counted using Coulter Counter Z1 (Bechman Coulter, Fullerton, CA). All conditions were performed in duplicate, and all experiments were performed three times.

### Adhesion assay

96-well round-bottomed plates were coated with human FN (20 μg/mL) overnight at 4°C, washed three times with PBS and blocked with 0,1 % BSA-PBS for 1 hour at room temperature, and then washed three times with Hanks’ Balanced Salt Solution (HBSS) (Sigma-Aldrich). Cells were labelled with acetoxymethyl ester-2′,7′ bis-(2-carboxyethyl)-5-(and-6)-carboxyfluorescein (BCECF-AM) (Sigma-Aldrich), for 1 hour in room temperature, and washed three times with HBSS before stimulated with cytokines as indicated for 60 minutes (5^*^10^4^ cells/well). Non-adherent cells were washed away by carefully pipetting with HBSS. Adherent cells were lysed with 1 % Triton-X. Fluorescence level at 538 nm was read by Victor^3^ plate reader and Wallac 1420 Work Station software. Adherent cells were calculated as percent of signal before washing. All conditions were performed in six parallel readings, and each experiment was performed three times.

### Flow cytometry

For intracellular flow cytometry, cells (1^*^10^6^) were fixed and permeabilized (Fix & Perm®Cell Fixation & Cell Permeabilization Kit) (ThermoFisher Scientific, Waltham, MA) according to manufacturer´s instructions. Cells were then incubated with antibody for 20 minutes in room temperature, and then washed and resuspended in 0,1% BSA-PBS. For surface proteins, cells (1^*^10^6^) were incubated with primary antibody and secondary antibody on ice. Flow cytometry was performed with BD LSRII Flow Cytometer.

### Calcium flux

The method for measuring Ca^2+^ flux was adopted from [40, 41]. Cells (1^*^10^6^) were washed in PBS (calcium-free), stained with 1 μM Fura Red-AM (Invitrogen) for 30 min at 37°C, washed again and resuspended in PBS. Emission was obtained with Qdot-700 and PE-Cy5-5 filter. Background signal was recorded for 20 seconds before the tube was removed and SDF-1α (100 ng/mL) or Cell Stimulation Cocktail (CSK) (500X) (eBioscience, San Diego, CA) 1:500 was added. Data, including the ratiometric Fura Red Ratio between Qdot-700 and PE-Cy5-5 and the percentage of responding cells, were analyzed with FlowJo v. 10.1.5 (FlowJo, LLC, Ashland, OR).

### Confocal microscopy

Cells were allowed to adhere to Poly-L-lysine (0,01 %), and then fixed with 4 % paraformaldehyde before permeabilized with PBS, 5 % human serum and 0,5 % saponin. PRL-3 was detected with directly conjugated PRL-3-Alexa 488 antibody and the nucleus was stained with Hoechst 33342 (ThermoFisher). Confocal imaging was performed using the Leica SP8 STED 3D microscope (Wetzlar, Germany). Imaris (Bitplane, Zurich, Switzerland) was used for imaging editing.

### Statistics

Statistical significance was determined using Student’s *t*-test in IBM SPSS Statistics ver. 24.

### Ethics

The use and storage of patient samples was approved by the Regional Ethics Committee (Approval #28/99 and 2015/700-03), and all patients gave informed consent.

## SUPPLEMENTARY MATERIALS FIGURES AND TABLES


