# Supplementary material for: Phosphatase of regenerating liver-3 is expressed in acute lymphoblastic leukemia and mediates leukemic cell adhesion, migration and drug resistance
Source: Oncotarget. 2017 Dec 13;9(3):3549–61. doi: 10.18632/oncotarget.23186 (PMC5790482; doi:10.18632/oncotarget.23186)
Supplement: Supplementary file 1 [file oncotarget-09-3549-s001.pdf]

Phosphatase of regenerating liver-3 is expressed in acute lymphoblastic leukemia and mediates leukemic cell adhesion, migration and drug resistance

SUPPLEMENTARY MATERIALS

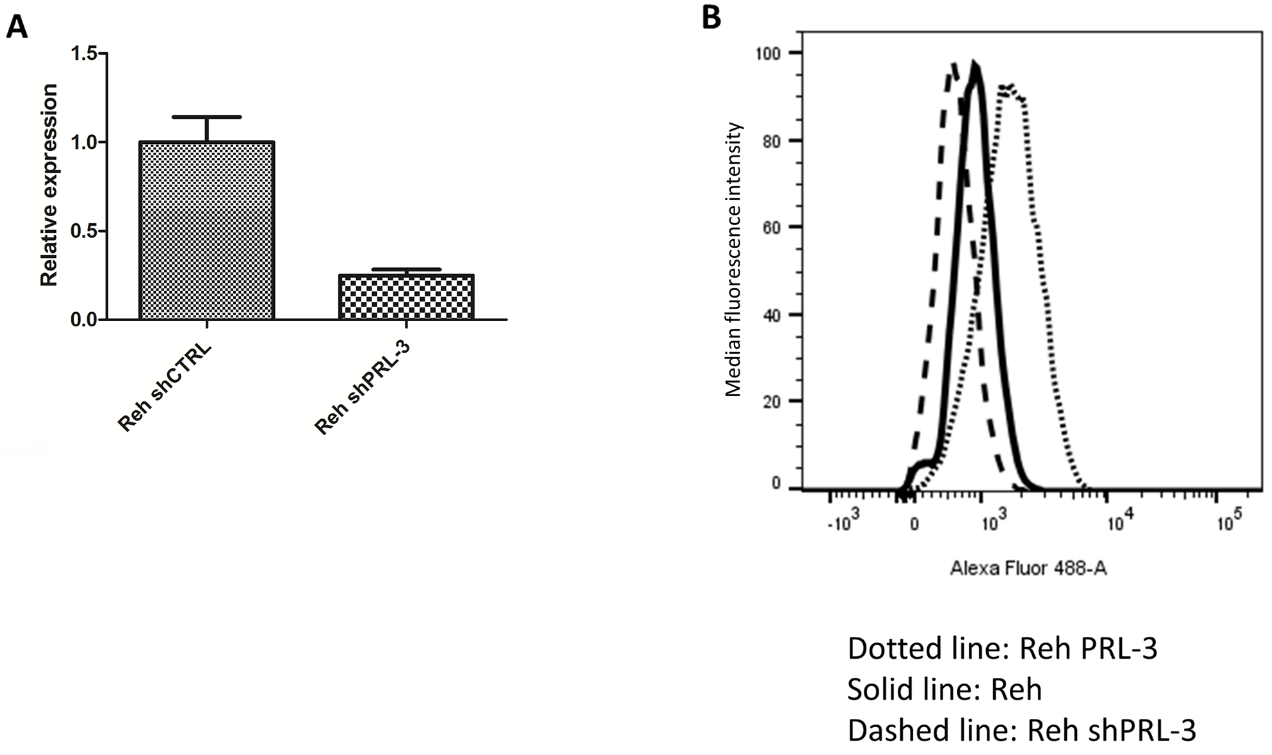

Supplementary Figure 1: (A) Knockdown of PRL-3 by shRNA was 75 % efficient as determinced by qRT-PCR. **(B)** PRL-3 knockdown (Reh shPRL-3) and doxycycline (1 µg/ml)-inducible PRL-3 expressing Reh (Reh PRL-3) compared to Reh. PRL-3 expression detected by flow cytometry. Median fluorescence intensity value is normalized.

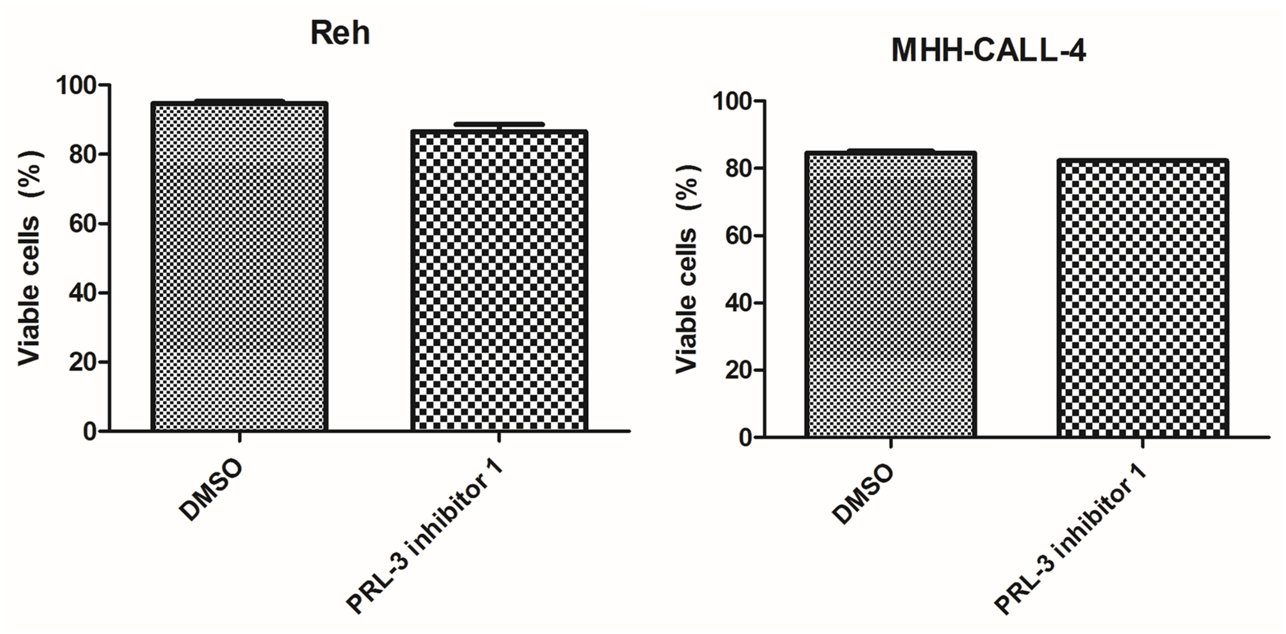

Supplementary Figure 2: Viability in Reh (left) and MHH-CALL-4 (right) after 48 hours treatment of PRL-3 inhibitor I, compared to DMSO control. Viability was measured by annexin-V by flow cytometry.

Supplementary Table 1: Clinical data from 18 adult B-ALL patients shown in Figure 1A

Age (years)	40 (21-48)
Gender (male/female)	10/8
White blood cells count (10^9/L)	8.5 (4.3-40)
Blast in bone marrow (%)	92 (83-96)
Blast in peripheral blood (%)	30 (10-90)

Values shown as median (interquartile range) with exception of gender (absolute count).

Supplementary Table 2: Clinical data from 12 pediatric B-ALL patients shown in Figure 1B

Age (months)	65 (26-130)
Gender (male/female)	5/7
B cells (median fluorescence intensity)	233 (162-305)
B-ALL cells (median fluorescence intensity)	310 (271-384)
White blood cells (10^9/L)	11.95 (5.4-28)
Blast in bone marrow (%)	79 (72-89)
Blast in peripheral blood (%)	46 (33-67)
Blast in central nervous system (%)	0 (0-4.8)

Values shown as median (interquartile range) with exception of gender (absolute count).
